# Migraine Treatment: Towards New Pharmacological Targets

**DOI:** 10.3390/ijms241512268

**Published:** 2023-07-31

**Authors:** Marcello Silvestro, Luigi Francesco Iannone, Ilaria Orologio, Alessandro Tessitore, Gioacchino Tedeschi, Pierangelo Geppetti, Antonio Russo

**Affiliations:** 1 Headache Centre, Department of Advanced Medical and Surgical Sciences, University of Campania “Luigi Vanvitelli”, 80138 Naples, Italy; ilaria.orologio@hotmail.it (I.O.); alessandro.tessitore@unicampania.it (A.T.); gioacchino.tedeschi@unicampania.it (G.T.); 2 Advanced MRI Neuroimaging Centre, Department of Advanced Medical and Surgical Sciences, University of Campania “Luigi Vanvitelli”, 80138 Naples, Italy; dottor.russo@gmail.com; 3 Headache Centre and Clinical Pharmacology Unit, Careggi University Hospital Florence, 50134 Florence, Italy; luigifrancesco.iannone@unifi.it (L.F.I.); pierangelo.geppetti@unifi.it (P.G.)

**Keywords:** CGRP, PACAP, VIP, TRP channel, adrenomedullin, amylin, KATP receptor, BKCa receptor, purinergic pathway

## Abstract

Migraine is a debilitating neurological condition affecting millions of people worldwide. Until a few years ago, preventive migraine treatments were based on molecules with pleiotropic targets, developed for other indications, and discovered by serendipity to be effective in migraine prevention, although often burdened by tolerability issues leading to low adherence. However, the progresses in unravelling the migraine pathophysiology allowed identifying novel putative targets as calcitonin gene-related peptide (CGRP). Nevertheless, despite the revolution brought by CGRP monoclonal antibodies and gepants, a significant percentage of patients still remains burdened by an unsatisfactory response, suggesting that other pathways may play a critical role, with an extent of involvement varying among different migraine patients. Specifically, neuropeptides of the CGRP family, such as adrenomedullin and amylin; molecules of the secretin family, such as pituitary adenylate cyclase-activating peptide (PACAP) and vasoactive intestinal peptide (VIP); receptors, such as transient receptor potential (TRP) channels; intracellular downstream determinants, such as potassium channels, but also the opioid system and the purinergic pathway, have been suggested to be involved in migraine pathophysiology. The present review provides an overview of these pathways, highlighting, based on preclinical and clinical evidence, as well as provocative studies, their potential role as future targets for migraine preventive treatment.

## 1. Introduction

Migraine is a complex and debilitating neurological disorder affecting millions of people worldwide, ranked as the first cause of life lived with disability in young women due to its considerable impact on personal well-being [1]. It is a chronic disease characterized by recurrent episodes of moderate-to-severe headaches, accompanied by neurovegetative symptoms, such as nausea; vomiting; and hypersensitivity to light, sound, and smell, as well as intolerance to physical activity [2]. The hallmark of migraine treatment is represented by approaches aimed to prevent future attacks based on lifestyle modifications, to avoid identified triggers or predisposing factors, as well as on preventive medications, and to reduce the frequency and severity of migraine attacks [3]. Until five years ago, preventive migraine treatments were based on ‘repositioning drugs,’ molecules with different and pleiotropic targets, developed for other indications and discovered by serendipity to be rather effective in migraine prevention (i.e., β-blockers, antiepileptics drugs, antidepressants, and calcium channel antagonists), although burdened by tolerability issues leading to low treatment adherence [4]. However, the significant progresses in unravelling the pathophysiology of migraine attacks allowed the identification of putative specific targets for preventive migraine treatments, such as the calcitonin gene-related peptide (CGRP) pathway [5,6]. Indeed, the advent of CGRP-monoclonal antibodies (CGRP-mAbs), or more recently of gepants, sets up, for the first time, a specific ‘ad-hoc’ approach against neuropeptides with a well-known role in migraine pathophysiology, representing a turning point in migraine preventive treatment due to the high efficacy, safety, and tolerability [7].

**Figure 1:**
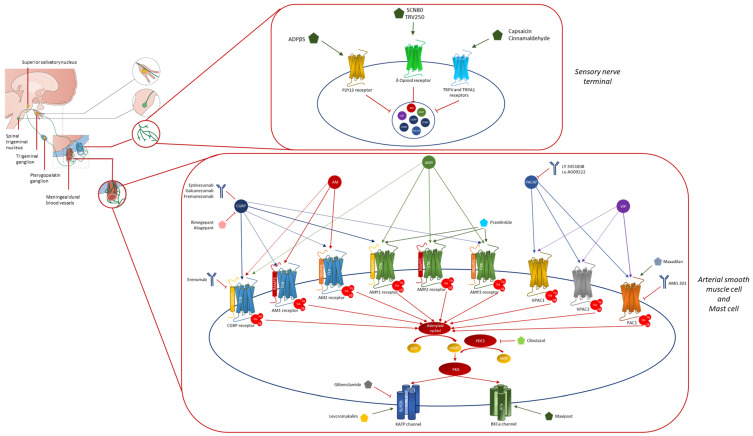
Schematic and simplified graphical representation of the pathways involved in migraine pathophysiology that might represent future targets for preventive treatments. All the pathways, activated by the different molecules, converge in a common intracellular downstream determinant where the second messenger cAMP finally leads to the opening of KATP and BKCa channels. Abbreviations: AC, Adenylate Cyclase; AM, Adrenomedullin; AMP, Adenosine Monophosphate; AMY, Amylin; BKCa, Big Conductance Calcium-Activated Potassium Channel; ATP, Adenosine Triphosphate; cAMP, Cyclic Adenosine Monophosphate; CGRP, Calcitonin Gene-Related Peptide; CLR, calcitonin like receptor; CTR, calcitonin receptor; KATP, Adenosine Triphosphate-Sensitive Potassium Channel; Kir, inward rectifier K+ channel; PAC1, Pituitary Adenylate Cyclase-Activating Polypeptide Type 1 Receptor; PACAP, Pituitary Adenylate Cyclase-Activating Polypeptide; PKA, Protein Kinase A; PDE3, phosphodiesterase 3; RAMP, receptor activity-modifying proteins; SUR, sulfonylurea receptor subunits; TRPA1, Transient Receptor Potential Ankyrin; TRPV, Transient Receptor Potential Vanilloid; VIP, Vasoactive Intestinal Polypeptide; VPAC, Vasoactive Intestinal Polypeptide Receptor.

Nevertheless, despite the revolution brought by CGRP-mAbs and gepants in migraine armamentarium, a significant percentage of patients (close to 40%) still remains burdened by an unsatisfactory response, suggesting that other molecules or pathways may play a critical role, with an extent of involvement varying among different migraine patients or, possibly, migraine attacks [8,9]. Indeed, both preclinical observations and provocative studies have widely demonstrated that the administration of various neuropeptides, other than CGRP, is able to trigger migraine-like attacks in a non-neglectable percentage of patients [10]. These findings have spurred research towards the identification of new pharmacological targets in order to develop other specific drugs (See Figure 1 and Table 1 for further information). 

In this review, we consider neuropeptides and pathways suggested by preclinical and clinical evidence, as well as provocative studies, to be involved in migraine pathophysiology, highlighting their role as potential future targets for migraine preventive treatment. Although it is not excluded that in the future other pathways will be demonstrated to be involved, we have considered all the molecular pathways with a demonstrated (also marginal) role in migraine pathophysiology.

## 2. Historical Background of Migraine Treatments 

Since the late 19th century, with the employment of ergotamine, a molecule derived from the ergot fungus, several drugs have enriched the migraine preventive armamentarium, such as tricyclic antidepressants, beta-blockers, calcium channel blockers, and anticonvulsants. As it can be easily guessed, the so-called “repositioning drugs” are quite different molecules, developed for other indications and identified by serendipity to be effective as preventive treatment of migraine based on mechanisms of action that are not always fully understood. Moreover, these preventive migraine medications, burdened by tolerability issues (due to highly pleiotropic targets) and low effectiveness (due to no specific mechanisms of action), frequently make the health-care of patients with migraine challenging and unpredictable. Indeed, repositioning preventive medications, when not contraindicated, are often overloaded by several bothersome adverse events [11]. Furthermore, even when repositioning drugs is well tolerated, a significant percentage of patients may not adequately respond (or not respond at all). Therefore, preventive treatments switches become necessary, finally resulting in a “trial and error process”, experienced as frustrating for both physicians and patients and leading to the well-known low adherence to therapeutic strategies (approximately 30%) observed in the migraine patients population [4,12].

In this scenario, the advent of CGRP-mAbs and the even more recent gepants, has represented a turning point, setting up a specific pharmacological strategy targeting the CGRP pathway, known to play a momentous role in migraine pathophysiology. Indeed, the CGRP capability to trigger a migraine-like headache when administered intravenously, along with the increased level of CGRP in the jugular blood, as well as in serum, cerebrospinal fluid, and saliva during spontaneous migraine attacks (followed by level normalization after effective triptans administration), are only a few among the numerous pieces of evidence strongly supporting the relevance of CGRP in the migraine mechanism [13].

On the other hand, as a proof-of-concept, since the eptinezumab phase 2 trial in patients with migraine, a plethora of subsequent clinical trials have demonstrated that CGRP-mAbs and second-generation gepants are effective, safe, and tolerable as preventive treatments in both patients with episodic and chronic migraine [7,14].

Nevertheless, approximately 40% of patients with migraine have demonstrated an inadequate response to drugs targeting the CGRP pathway that could tie in with the percentage of migraine patients (about 35–40%) in which the intravenous administration of CGRP is not able to induce migraine-like attacks. The lack of response to drugs targeting the CGRP pathway can be underlined by different reasons, such as (i) the CGRP pathway could be insufficiently blocked; (ii) CGRP ligands or receptors could be sufficiently blocked, but other peptides could activate the CGRP receptors, or the CGRP ligand could work on different receptors; (iii) the CGRP pathway could be sufficiently blocked, but migraine may be induced via other signaling pathways [15].

Starting from these insights, preclinical and clinical evidence, further supported by provocative studies, has demonstrated the involvement of numerous neuropeptides in migraine pathophysiology, putatively representing targets for future migraine preventive treatments.

## 3. The Vasoactive Intestinal Polypeptide (VIP)/Glucagon/Secretin Family 

The vasoactive intestinal polypeptide (VIP)/glucagon/secretin family is a group of related peptides, including, among others, pituitary adenylate-cyclase-activating polypeptide (PACAP), gastric inhibitory polypeptide (GIP), glucagon, and secretin [16]. The members of the VIP/glucagon/secretin family share sequence similarities and have overlapping functions in numerous physiological processes. They are involved in the regulation of metabolism, blood flow, immune responses, gastrointestinal functions, and neuronal signaling [16]. The role in migraine pathophysiology of two of them, PACAP and VIP, has been extensively studied.

### 3.1. PACAP

PACAP is a neuropeptide, first discovered in 1989, able to stimuli the production of cyclic adenosine monophosphate (cAMP) in the pituitary gland, hence its name [17]. In the last few decades, a growing body of scientific literature pointed out the role of PACAP in migraine pathophysiology [18]. PACAP can be found in two different isoforms with similar affinity and functions: PACAP38, representing approximately 90% of the total PACAP in mammalian tissues, and its truncated isoform, PACAP27 [19]. The PACAP38 segment 28-to-38, differentiating PACAP 38 from PACAP27, is recognized by the blood–brain barrier transporter to be transported into the brain tissues. The PACAP38 half-life is of less than 5 min in human plasma in vitro, while the PACAP27 half-life is 45 min. 

PACAP molecules exerts their effects at four different receptors: PAC1, VPAC1, VPAC2, and the orphan MrgB3 receptor (in rodents) or MrgX2 receptor (in humans). VPAC1 and VPAC2 have equal affinity for PACAP38 and VIP ligands, while the PAC1 receptor has higher affinity (300- to 1000-fold higher) for PACAP38 than for VIP [20,21]. 

Similarly to CGRP, PACAP is involved in various biological functions, including neurotransmission, vasodilation, intestinal motility, cell proliferation, differentiation, neuroprotection, reproduction, and immunity [22]. Specifically, in the nervous system, PACAP38 acts as a hormone, a neurotransmitter, and a neuromodulator, and its receptors are found in several important structures involved in pain perception and modulation as perivascular fibers, trigeminal and sphenopalatine ganglia, and the trigeminal nucleus caudalis [23]. Interestingly, the locations of PACAP and CGRP neuropeptides and their receptors are overlapping and well-positioned to contribute to peripheral and central actions in migraine. Indeed, both peptides and their receptors are found in multiple areas of the peripheral and central nervous system relevant to migraine. Specifically, within the peripheral nervous system, it has been showed that while CGRP is predominantly expressed in sensory neurons of the dorsal root and trigeminal ganglia, PACAP is mainly expressed in extracranial parasympathetic sphenopalatine ganglion [24]. Specifically, in the trigeminal ganglion, besides neurons expressing only CGRP (about 68%), there are 32% of neurons expressing PACAP38 [25]. PACAP receptors (PAC1 receptors) are found on trigeminal nerve endings, and activation of these receptors can lead to the release of CGRP, contributing to neurogenic inflammation and pain sensation, both of which are involved in migraine attacks. Within the CNS, both peptides are found in the thalamus and hypothalamus, as well as second-order neurons of the trigeminal nucleus caudalis. While the different PACAP receptors are widely expressed in the trigeminal ganglion and adjacent glial cells, VPAC1–2, but not PAC1, are expressed on intracranial arteries. 

The role of PACAP38 in migraine pathophysiology is supported by increased plasma levels observed during spontaneous migraine attacks compared to the interictal phase, as well as by provocative studies [26,27]. The latter showed that intravenous administration of PACAP38 for 20 min can induce headache in healthy volunteers and delayed migraine-like attacks in 58–73% of patients with migraine without aura (MwoA), although the 20 min intravenous infusion of PACAP27 is able to provoke migraine-like attacks in the 55% of patients with migraine [28,29,30,31]. Interestingly, despite a provocation rate similar to CGRP, PACAP38-induced migraine attacks were more frequently accompanied by premonitory symptoms typically experienced by migraine patients (i.e., fatigue, yawning, neck stiffness, hunger or food cravings, mood swings, and poor concentration), but with a longer duration of flushing [31]. It could be hypothesized that the ability to enter the central nervous system (CNS), as well as the high concentration of PACAP38 and its receptors in the hypothalamus, known to be involved in the prodromal phase of the migraine cycle, could explain the higher incidence of premonitory symptoms administering PACAP38 compared to CGRP [24]. 

The PACAP38-induced migraine attacks are potentially mediated by its vasodilatory properties, along with the meningeal mast cells degranulation [18,29]. Although there is still not converging evidence about the PACAP38 receptor able to mediate vasodilation, VPAC1–2 receptors are the most likely involved, considering that PAC1 receptor antagonist is unable to inhibit PACAP38-induced vasodilation in “in vivo” studies [32]. 

Interestingly, after intravenous PACAP38, a prolonged dilatation has been observed only in extracranial and meningeal arteries, but not in cerebral arteries, probably because, in spite of the described transport mechanism, PACAP38 is only minimally able to pass the blood–brain barrier (0.053%) [33]. Therefore, it can be argued that the PACAP site of action is mainly outside of the blood–brain barrier and most probably associated with vasodilation of the meningeal arteries. The role of vasodilation in PACAP38-induced migraine attacks seems to be further enforced by the ability of sumatriptan, a well-known anti-migraine drug with vasoconstrictor properties, to prevent PACAP38-induced migraine attacks (induction rate 15% after sumatriptan vs. 42% after placebo) [34]. Nevertheless, the pre-treatment with sumatriptan (but not ketorolac) is able to prevent PACAP38-induced migraine attacks without affecting PACAP38-induced arterial dilation, while post-treatment with ketorolac, but not sumatriptan, attenuated PACAP38-induced headache [35]. These findings suggest that PACAP38-induced headache in healthy volunteers can be attenuated by sumatriptan independently from vasoconstriction, with time-dependent preventative effects probably inhibiting central sensitization by blocking peripheral signal transmission from the meningeal nociceptors [35]. Similarly, since ketorolac antinociceptive effects may depend, at least partially, on the inhibition of inflammatory mediators released by mast cells (without acting on arterial dilation), the pronounced skin flushing (alleviated by anti-histaminergic drugs) may be underpinned by mast cells degranulation induced by intravenous infusions of PACAP38 in humans [36]. However, a recent study has downsized the role of mast cell degranulation in PACAP38-induced migraine-like attacks, reporting that pretreatment with H1-antihistamine and clamastine (anticholinergic drug) was unable to prevent attacks [37]. Similarly, no change in peripheral plasma levels of inflammatory mast cell mediator tryptase was observed after PACAP38 infusion in patients with MwoA [38]. However, it cannot be excluded that the timing and the site of collection might play a role in detecting altered plasma levels of mediators of mast cell degranulation, and it is unknown whether peripheral plasma may reliably reflect cranial release of mast cell mediators and, in turns, mast cell degranulation cannot be completely dismissed as a mediator of PACAP38-induced migraine attacks [39,40]. 

Also, considering the mast cell degranulating effect of PACAP38, the mainly involved receptors are matter of debate. Indeed, if on one hand, preclinical evidence failed to demonstrate the role of PAC1 receptors in mast cell degranulation, on the other hand, the MrgB3 receptor, corresponding in humans to MRGX2 receptor, seems to have a role, in particular in the long-lasting flushing and in delayed migraine attacks observed after PACAP38 infusion in humans [41].

Independently from the role of vasodilation and mast cell degranulation in the PACAP38-induced migraine attacks and the specific receptor involved, there is compelling preclinical evidence suggesting that the PACAP38 pathway is different from other migraine-provoking pathways [42,43]. Indeed, against the old hypothesis that PACAP and CGRP pathways are converging through the cAMP in the opening of adenosine triphosphate-sensitive potassium (KATP) channels, recent observation supports that PACAP38-induced hypersensitivity is not mediated via an increase in CGRP release, clearly highlighting the PACAP38 pathway as distinct from other migraine-provoking pathways, such as CGRP and the glyceryl trinitrate (the latter well-known as a CGRP-related) migraine trigger. For instance, a study on mice with genetically modified CGRP receptors (receptor activity-modifying proteins-1 knockout) showed that PACAP38 induced hind paw hypersensitivity and carotid artery vasodilation in receptor activity-modifying proteins-1 knockout mice, as well as in mice pre-treated with monoclonal antibody against CGRP. Similarly, monoclonal antibodies against PACAP38 did not improve CGRP-induced light aversion (as a surrogate for migraine-like photophobia) in mice, whereas CGRP-mAbs did not block light aversion induced by infusion of PACAP38 [44]. Therefore, PACAP antagonism has been clearly identified as a novel therapeutic target, and therefore, specifically considering preliminary provocation studies showing that the infusion of PACAP, but not of VIP, was able to induce migraine-like attacks, the attention was primarily focused on PAC-1 receptor (characterized by an extremely higher affinity for the PACAP compared to the VIP unlike the VPAC1 and VPAC2 receptors) [45]. Surprisingly, monoclonal antibodies targeting the PAC1 receptor failed to demonstrate therapeutic benefits in a phase 2 randomized study controlled with placebo [46]. On the other hand, since PACAP38 exerts its activity on other receptors in addition to PAC1, it cannot be excluded that monoclonal antibodies targeting the PACAP ligand may be more beneficial as a migraine preventive strategy, and there are anti-PACAP monoclonal antibodies targeting the ligand under development (i.e., LY 3451838, Lu AG09222) to date [47]. Very recently, a randomized, double-blind, parallel-group, single-dose, placebo controlled study demonstrated that Lu AG09222 reduced headache in healthy controls and inhibited concomitant cephalic vasodilation and increased heart rate induced by PACAP38 [48]. Similarly, positive results have been announced regarding the phase II study with Lu AG09222 in migraine prevention (HOPE trial), with a statistically significantly greater reduction in the number of monthly migraine days (MMDs) from baseline to weeks 1 to 4 of treatment in patients treated with the antibody anti-PACAP ligand compared to placebo in the absence of tolerability issues.

Even though some may be concerned about PACAP therapies side effects due to their multiple functions, no considerable adverse effects were reported in phase I and II clinical trials. 

### 3.2. VIP 

VIP is a member of the VIP/secretin/glucagon superfamily of peptides, structurally connected to PACAP, that serves various functions in the body. Its primary role is vasodilation, occurring in both systemic circulation and specific tissues, including the respiratory system, gastrointestinal tract, and the central and peripheral nervous systems [49]. However, it is involved in the regulation of different physiological processes, such as smooth muscle contraction, glandular secretion, immune responses, and inflammation [49]. Apart from its vasodilatory function, VIP also acts as a neurotransmitter and neuromodulator in cerebral parasympathetic perivascular nerve fibers and cranial parasympathetic ganglia [50]. The biological effects of VIP are mediated via VPAC1 and VPAC2 receptors and, to a much lesser extent, PAC1 receptors. VIP and its receptors are expressed in smooth muscle cells, neurons, and glial cells of trigeminal and sphenopalatine ganglia, and trigeminal nucleus caudalis [51]. VIP can only cross the blood–brain barrier to a very small extent. The putative role of VIP in migraine pathophysiology is still debated: on one hand, elevated VIP plasma levels have been reported in the cranial circulation of migraine patients with pronounced autonomic symptoms in the course of migraine attacks, but also in chronic migraine patients during the interictal period, when compared to both episodic migraine patients and healthy controls [52]. On the other hand, provocation studies with a 20 min infusion of VIP resulted in a short-lasting dilation of the superficial temporal and middle meningeal arteries in the absence of headache, in both healthy controls and patients with migraine [29,53,54]. VIP is the first substance that markedly dilates intra- and extracranial arteries; nevertheless, it does not induce migraine, further weakening the hypothesis of a purely vascular origin of migraine. However, more recently, a model of prolonged infusion of VIP (e.g., 2 h) has been proven to cause long-lasting dilation of the superficial temporal artery and delayed headache in both healthy volunteers and patients with migraine [55,56]. More specifically, a randomized, double-blind, placebo-controlled, 2-way crossover study demonstrated that 2 h VIP infusion induced the ignition of a migraine attack in 71% of patients compared with only 5% after placebo administration. It is noteworthy that, despite CGRP plasma levels elevation following prolonged VIP infusion, no correlation was found between CGRP levels and the occurrence of VIP-induced migraine-like attacks, supporting that VIP-induced migraine-like attacks are not mediated by CGRP release [57]. 

However, it remains to be delineated the receptors that specifically mediate VIP-induced vasodilation. Vasoactive peptides are able to activate receptors expressed on smooth muscle cells and upregulate intracellular cAMP, initiating a signaling cascade resulting in the opening of potassium channels and, in turns, vasodilation. It is unlikely that VIP-induced migraine-like attacks are mediated via PAC1 due to (1) the very low affinity of VIP for PAC1 receptor and (2) their debated presence in the cranial vasculature (while PAC1 receptors are expressed in the trigeminal and sphenopalatine ganglia, as well as the spinal trigeminal nucleus).

As proof of concept, maxadilan, a selective PAC1 receptor agonist, did not show vasodilator effects in intracranial and extracranial rat arteries [58]. Therefore, dilation of cranial arteries induced by VIP and PACAP is thought to be predominantly mediated by the prolonged stimulation of VPAC1 and VPAC2 receptors, leading to the opening of KATP channels or big-conductance calcium-activated potassium (BKCa) channels in the extracranial meningeal vasculature [59]. Whether prolonged extracranial artery dilation alone can explain VIP-induced migraine-like attacks or whether other mechanisms should be invoked is questionable to date. It cannot be excluded that VIP-induced migraine-like attacks may be at least partially mediated via mast cells-related neurogenic inflammation although a recent study conducted in sympathectomized rats, an experimental preclinical model of migraine, showed a modulating role of VIP in mast cell behavior [60]. More in depth, VIP is able to decrease both mast cells and c-Fos expression in the trigeminal nucleus efficiently modulating neurogenic inflammation of the dura by reducing the number of local mast cells induced by sympathectomy.

Altogether these data, along with the failure of a monoclonal antibody targeting the PAC1 receptor (AMG 301) in migraine prevention, raise some questions about the role of PAC1 receptor in the ignition of migraine-like attacks after both PACAP38 and VIP intravenous infusion, strongly suggesting the momentous role of VPAC1 and VPAC2 receptors as possible targets.

## 4. The Calcitonin/CGRP Family

The calcitonin/CGRP family is a group of peptides sharing structural similarities including calcitonin, CGRP, amylin, and adrenomedullin [61]. Beyond CGRP, both amylin and adrenomedullin have been extensively investigated to ascertain their involvement in migraine pathophysiology. Receptors for calcitonin peptide family consist of two G protein-coupled receptors as the calcitonin receptor (CTR), and the calcitonin like receptor (CLR), together with an accessory receptor activity-modifying proteins (RAMPs) [62]. These protein–protein interactions enable receptors with variable affinity for different combinations of calcitonin-family peptides.

### 4.1. Adrenomedullin

Adrenomedullin is a multifunctional endothelial peptide hormone first isolated in 1993 from the adrenal medulla, hence its name [63]. It exists in two different isoforms with almost overlapping affinity and functions: AM1, a 52-amino acid peptide, and AM2, a 53-amino acid peptide. Both isoforms are expressed at high concentrations by vascular endothelial cells, smooth muscle cells, and immune cells [64]. Adrenomedullin is widely distributed throughout the body and participates in various physiological processes, including vasodilation, bronchodilation, fluid homeostasis, angiogenesis, immune response, and hormone regulation [64]. Due to its diverse functions and widespread distribution, adrenomedullin has attracted considerable attention as a potential therapeutic target for several disorders, such as hypertension, retinopathy, and tumorigenesis.

The strong molecular relationship with CGRP suggests a role of adrenomedullin in migraine pathophysiology, likely mediated by its effects on vasodilation, neurogenic inflammation and pain perception and transmission [65]. 

Preclinical studies have shown that administration of adrenomedullin induces dilation of dural and pial arteries, reduces mean arterial blood pressure, and increases local cerebral blood flow [66]. However, these effects are inhibited by canonical CGRP-receptor antagonists. Nevertheless, nitric oxide and cAMP are involved in adrenomedullin-induced vasodilation, further implicating its role in migraine-related vasodilation [67]. Adrenomedullin exerts a proinflammatory, but also an anti-inflammatory, effect by inhibiting the production and release of pro-inflammatory cytokines, such as tumor necrosis factor-alpha (TNF-α) and interleukin-6 (IL-6), in various preclinical experimental models [68]. Furthermore, if on one hand adrenomedullin is reported to have pro-nociceptive functions, on the other hand it modulates pain processing at various levels, including the spinal cord and the trigeminal system [69]. Specifically, experimental preclinical investigations showed that the intrathecal administration of adrenomedullin can induce thermal hyperalgesia and mechanical allodynia in the rat formalin test [70,71]. Interestingly, mouse model knockout for adrenomedullin showed longer latency in the tail-flick test, measuring the spinal reflexes, and shorter latencies in the hotplate test, requiring encephalic processing, suggesting that adrenomedullin acts as a nociceptive modulator in spinal reflexes but has analgesic function at higher cognitive levels [72]. However, no studies have explored adrenomedullin-induced non-nociceptive migraine-like responses (e.g., light aversion).

Interestingly, while intravenous infusion of adrenomedullin did not cause headache attacks in healthy subjects, conflicting results were observed in patients with MwoA [73,74,75]. In a first crossover double-blind study conducted on 12 patients, no differences were observed in the percentage of patients reporting headache attacks, as well as in cerebral blood flow, following adrenomedullin or placebo administration [73]. However, the nocebo migraine response rate in this study (33.3%) was significantly higher than the average nocebo response rate in migraine provocation studies and may have therefore confounded the interpretation of results [74]. Nevertheless, a more recent study conducted on a larger sample of 20 patients using the same protocol showed a significantly higher percentage of headache attacks when patients were exposed to adrenomedullin compared to placebo (55% vs. 15%) but common and expected adverse events associated with adrenomedullin infusion (e.g., increased heart rate, palpitations, facial flushing, and heat sensations) may have unblinded the study [75]. 

It is also possible that adrenomedullin administration may act through the canonical CGRP receptor, as the estimated blood concentrations reached in these studies would likely activate, in addition to the AM1 and AM2 receptors, also CGRP receptor [76]. Notably, adrenomedullin-induced migraine-like attacks were similar in terms of time to onset and pain distribution to CGRP-induced migraine-like attacks. To clarify whether the canonical CGRP receptor mediates the response to adrenomedullin, further evaluation could be conducted to determine if a gepant, an antagonist of the CGRP receptor with very low affinity for AM receptors, is able to inhibit AM-induced migraine-like attacks. In conclusion, unlike the other neuropeptides, the involvement and the role of adrenomedullin in migraine attacks are less clear and needs further investigation before a clear-cut definition as a promising target in migraine pharmacological management.

### 4.2. Amylin

Amylin is a 37-amino acid peptide that belongs to the CGRP peptide family, sharing significant amino acid sequence identity with CGRP [77]. This neuroendocrine hormone is secreted alongside insulin from pancreatic β-cells in response to food intake. It acts in the brain to promote satiation, signaling the end of a meal, and lowers blood glucose levels by decreasing glucagon secretion [78]. Pramlintide, an amylin mimetic, is an approved drug for the treatment of insulin-requiring diabetes and has also been studied as a potential treatment for obesity in clinical trials [79].

Amylin expression extends beyond pancreatic cells and can be found in the dorsal root ganglia, as well as neurons of the trigeminal ganglia [80]. However, antibodies used to detect amylin in the trigeminal ganglia also recognize CGRP due to the high sequence similarities between these two neuropeptides. Amylin binds to at least three distinct receptors with high affinity: AMY1, AMY2, and AMY3 [61]. These receptors consist of core CTR in combination with one among RAMP1, RAMP2, or RAMP3. Interestingly, the AMY1 receptor, which contains the RAMP1 subunit, can also be activated by CGRP [61,62]. However, while amylin is potent at the amylin receptor, it has weak activation at the CGRP receptor, contrariwise CGRP activates both receptors with similar potency. Notably, medications like gepants and erenumab effectively antagonize the CGRP receptor and the AMY1 receptor and that is the mechanism proposed to explain differences in adverse effects between monoclonal antibodies targeting the CGRP ligand versus the CGRP receptor and to provide a rationale for observed differences when switching between these molecules [15].

The potential role of amylin in pain has been explored in preclinical studies, which indicate that amylin can induce pro-nociceptive and migraine-like behaviors in rodents. For example, mice knock-out for amylin showed reduced nociceptive behaviors in response to chemical pain stimuli (formalin injections) while amylin in female mice (but not males) decreases mechanical pain thresholds, increases light aversion, and induces squint response while reduce thermal threshold via intracerebroventricular injection [81,82,83]. The pain behavior following amylin was consistently reversed when amylin antagonists, such as AC187 and rat Amy8–37, were administered [84,85]. Therefore, it can be argued that the effects of amylin strongly depend on factors, such as gender, route of administration, or specific nociceptive pathways investigated.

Contrariwise, studies examining amylin in primary headache disorders suggest a potential pro-nociceptive role. Elevated levels of circulating amylin have been found in patients with chronic and, to a lesser extent, episodic migraine, independent of metabolic status and obesity, suggesting a significant role in migraine pathophysiology [86]. Indeed, a double-blind crossover study demonstrated that the percentage of patients with migraine reporting headache attacks or migraine-like attacks after pramlintide infusion was not significantly different from CGRP infusion [87]. Specifically, pramlintide was able to induce headache in 88% of patients and a migraine-like attack in 41% of patients (compared with 97% and 56% respectively with CGRP infusion). Pramlintide, like amylin, is a weak agonist of the canonical CGRP receptor (CLR/RAMP1), with even less activity at the adrenomedullin receptors. This suggests that the CTR/RAMP complexes, particularly AMY1, mediate pramlintide-induced migraine-like attacks. Interestingly, some patients respond only to CGRP, while others respond only to pramlintide. Similarly, pramlintide administration did not elevate circulating CGRP levels, and the peak plasma concentration of pramlintide was insufficient to activate the CGRP receptor, indicating that pramlintide-induced migraine-like attacks are likely independent of the involvement of CGRP pathway. Although the understanding of amylin’s functions and its potential contribution to migraines and pain disorders is still evolving, altogether these findings tend to highlight the possibility of different involvement of CGRP and amylin pathways among different migraine patients, providing a rationale for the development of molecules that selectively target amylin pathway.

## 5. Potassium Channels

Targeting each signaling pathway separately has proven to be only partially successful, suggesting that a common downstream determinant may represent a new target potentially able to revolutionize the migraine preventive treatment. In this context, it is noteworthy that the pathways of CGRP, PACAP, adrenomedullin, and amylin, but also nitric oxide, converge to increase cAMP or cyclic guanosine monophosphate (cGMP) levels leading to the final opening of KATP or BKCa channels with consequent cellular potassium efflux and, in turns, membrane hyperpolarization and tissue specific cellular response.

### 5.1. KATP Channel

KATP channels are large hetero-octameric transmembrane protein complexes constituted by four pore-forming inward rectifier K+ channel subunits (e.g., either Kir6.1 or Kir6.2) and four regulatory sulfonylurea receptor subunits (e.g., SUR1, SUR2A, or SUR2B) [88]. Firstly described in cardiac muscle cells, KATP channels are also found in the pancreas, skeletal muscle cells, smooth muscle cells, and nervous system [89]. KATP channels are characterized by structural and functional diversity determined by distinct combinations of Kir and SUR subunits. Kir6.2/SUR1 KATP channels are expressed in pancreatic β-cells, peripheral nervous system (e.g., trigeminal ganglia) and CNS (e.g., trigeminal nucleus caudalis), while Kir6.2/SUR2A subtypes are exclusively found in cardiac cells [90]. However, the most relevant in migraine pathophysiology seems to be Kir6.1/SUR2B constituting the major KATP channel in vascular smooth muscle cells, as well as in trigeminal ganglia and in trigeminal nucleus caudalis. The KATP channels are activated by Mg^2+^-bound nucleotides and adenosine diphosphate (ADP, which act on the SUR subunit, and inhibited upon binding of intracellular ATP to the Kir6 subunit. Thus, KATP channels are open during states of low metabolic activity (e.g., low levels of ATP/high levels of ADP) resulting in plasma membrane hyperpolarization [91]. The ability to couple cellular metabolic state (ATP/ADP ratio) to the electrical activity of the cell membrane is critical in numerous physiological processes and is a key feature of KATP channels [92]. 

Several KATP channel openers (such as nicorandil, minoxidil, pinacidil, and diazoxide) are used in clinical practice due to vasodilating effects in angina and hypertension, as well as alopecia, although burdened by headache, one of the most reported side effect due to the fact that KATP channels (particularly Kir6.1/SUR2B subtypes) are also expressed in the middle meningeal artery [93]. 

Moreover, while intravenous administration of levcromakalim, a KATP channel opener, induces headache and middle meningeal artery dilation in healthy volunteers, reversed by sumatriptan injection, it provokes migraine-like attacks in 100% of patients with migraine and migraine with aura attacks in the 59% of patients with migraine with aura [94,95]. These observations led to investigations on blockage of KATP channels by glibenclamide (a nonspecific KATP channel blocker belonging to the second generation of sulfonylurea used in diabetes mellitus) in order to ascertain its reliability as a putative approach for migraine prevention [96,97]. Actually, although the efficacy of glibenclamide in reversing the vasodilation related to CGRP, PACAP, or prostacyclin and inhibiting cutaneous hypersensitivity in female mice injected with levcromakalim or glyceryl trinitrate was demonstrated in preclinical studies, it failed to prevent headache attacks induced by levcromakalim, PACAP38, or CGRP in humans, being only able to delay the onset of levcromakalim-induced headache [97,98,99]. It could be argued that headache attacks are mediated by the activation of specific isoforms of KATP channels sulfonylurea receptor subunits, and KATP blockers more selective than glibenclamide are needed to further elucidate the role of potassium channels in migraine pathophysiological mechanisms. For instance, the SUR2B subunit, widely expressed in the trigeminovascular system, may represent a valuable future therapeutic target for migraine treatment.

### 5.2. BKCa

BKCa channels are octameric membrane protein complexes consisting of four pore-forming subunits (α) associated with four accessory subunits (β) regulating channel gating behavior [100]. Four distinct β-subunits (β1–β4) have been discovered. The β2 and β3 subunits share sequence similarities with β1, but unlike β1 and β4, which favor the active conformation, β2 and β3 promote a fast-inactive conformation in BKCa channels [101]. The β1 subunit is primarily expressed in smooth muscle and some neurons of trigeminal ganglion and trigeminal nucleus caudalis, while the β4 subunit is highly expressed in the whole brain [102,103].

BKCa channel openers, such as andolast and cilostazol, have been investigated for the treatment of bronchial asthma and intermittent claudication, respectively and both were associated with frequent adverse events, including headache [86]. Specifically, cilostazol is known to induce headaches in healthy volunteers, as well as migraine-like attacks in patients with migraine [104,105]. To confirm the role of BKCa channels in migraine pathophysiology, MaxiPost, a positive modulator of BKCa channels, with high lipophilic properties facilitating its crossing of the blood–brain barrier, has been used to systematically explore headache following BKCa channel activation [104,105]. Specifically provocative studies have shown that 20 min continuous intravenous administration of 0.05 mg/min MaxiPost induced headache attacks in 90% of healthy controls and in 100% of patients with MwoA [104,105]. Interestingly, 95% of patients with migraine developed migraine-like attacks. Long-lasting vasodilation resulting in mechanically induced sensitization of perivascular trigeminal afferents, as well as potassium (K+) outflow and extracellular accumulation, resulting in chemically induced sensitization of perivascular trigeminal afferents, have been proposed as mechanisms for BKCa channel activation-induced migraine attacks [104,105].

Several nonselective BKCa channel blockers, such as iberiotoxin and paxilline, have been developed, but they are not approved for clinical use due to the risk of severe on-target or off-target adverse events since BKCa channels are distributed throughout the body [106,107]. Therefore, selective blockers against β1 or β4 subunits, known to be expressed in migraine-related structures, are required in order to develop effective therapeutic molecules with good safety and tolerability profiles [108,109].

## 6. Purinergic System

The purinergic system is a complex signaling pathway involving the detection, release, and response to purine nucleotides and nucleosides, such as adenosine and ATP involved in various physiological and pathological processes [110,111]. Indeed, the purinergic system is involved in the regulation of cardiovascular function, immune responses, gastrointestinal motility, and renal function, as well as in inflammation and pain perception [110,111]. Additionally, it plays a critical role in the regulation of neurotransmission in the central and peripheral nervous systems, where ATP and adenosine act as neuromodulators and participate in synaptic transmission and plasticity [112]. There are 19 purinergic receptors known to date, sub-divided into two main groups: adenosine receptors (P1 receptors) and purine/pyrimidine nucleotide (ATP, ADP, UTP, UDP) receptors (P2 receptors) [113,114]. More in depth, there are four subtypes of P1 receptors, namely A1, A2A, A2B, and A3, and fifteen P2 receptors represented by two families: P2X ionotropic ligand-gated ion channel receptors and P2Y metabotropic G protein-coupled receptors. ATP may act as a neurotransmitter in non-adrenergic non-cholinergic (NANC) nerves supplying gut and bladder or as a co-transmitter in sympathetic nerves (along with noradrenaline and neuropeptide Y), parasympathetic nerves (along with acetylcholine and vasoactive intestinal peptide), in enteric NANC inhibitory nerves (with nitric oxide and VIP), and in sensory–motor nerves (along with CGRP and substance P). Both groups of receptors, namely P1 and P2, have been explored as therapeutic targets for several conditions. Among these, the agonist adenosine (which binds to P1 receptors) is used to treat supraventricular tachycardia whereas antagonists of P2Y12 receptor (mainly distributed on platelets), particularly clopidogrel and ticlopidine, are currently used as anti-platelet agents [115].

The involvement of purinergic signaling in migraine pathophysiology is strongly supported by the higher adenosine blood concentration in the course of migraine attacks, as well as by the ability of adenosine or dipyridamole (an adenosine uptake inhibitor) to induce headache or migraine-like attacks when administered intravenously in 78% of patients with migraine [116]. 

Interestingly, adenosine, through P1 receptors, can play a dual role in migraines since it could cause both sensitization of the trigeminovascular system and its inhibition [117]. More specifically, the stimulation of adenosine A1 receptors (expressed in the trigeminovascular system, including the trigeminal ganglion and trigeminal nucleus caudalis) inhibits trigeminovascular system by reducing neuronal firing from the trigeminal nucleus and decreasing the CGRP release. On the contrary, the stimulation of A2A and A2B receptors, located in vascular smooth muscle cells and in pre- and postsynaptic nerve terminals, causes dural vasodilation, leading to stimulation of the trigeminovascular system [117]. The proof of the concept comes from preclinical studies using selective A1 receptor agonists GR79236 and GR190178, able to inhibit trigeminovascular system by acting directly on the trigeminal nucleus and inhibiting the release of CGRP, in the absence of vasoconstriction [118]. Unfortunately, these molecules due to their off target effects on heart and autonomic nerves frequently induce bradycardia and hypotension making this approach not affordable in clinical practice. 

On the other hand, P1 and P2 receptors, widely expressed in the CNS, particularly on the neurons and glial cells, as well as on the smooth muscles and endothelium in the vascular system, seem to be similarly involved in migraine pathophysiology [116]. In particular, the activation of P2X receptors on smooth muscles by ATP (along with noradrenaline as co-transmitter) induces vasoconstriction being released by perivascular sympathetic nerves and damaged endothelial cells. Furthermore, ATP activates the P2X and P2Y receptors on the endothelial cells, which release endothelium-derived relaxing factor (EDRF) into the blood, leading to vasodilation and resulting in reactive hyperemia and pain. Moreover, P2Y13 and P2X3 receptors are expressed also in both human trigeminal ganglion and middle meningeal artery [119]. The administration of an agonist of P2Y13 receptors (e.g., ADPβS) can cause middle meningeal artery contraction in vitro, reduced dural artery vasodilation following periarterial electrical stimulation in vivo, and reduction of CGRP release from both the dura and the trigeminal ganglion in situ [119]. On the other hand, the administration of an agonist of P2X3 receptors (e.g., abmetATP) causes both CGRP release and middle meningeal artery dilation, making ATP-gated P2X3 receptor antagonists promising candidates for antimigraine drug development, such as AF-219 and A-317491 characterized by favorable pharmacodynamic and pharmacokinetic properties worthwhile for further exploration in clinical trials [120,121]. However, the complexity of the purinergic signaling system and the lack of suitable animal models pose limitations in translating this research into clinical applications [116].

## 7. Opioid System

The opioid system plays a central role in pain perception and modulation, but also in mood and the sense of well-being [122]. There are four major subtypes of opioid receptors –µ, δ, and κ-opioid receptors, and opioid receptor-like 1 (ORL-1)/nociception receptor [123]. Several endogenous substances exert their effect on these receptors. Among these, enkephalins are endogenous ligands with high affinity for the δ receptor, whereas endorphins preferentially activate the µ receptor, and dynorphins preferentially activate the κ-receptor. Herein, just for sake of completeness, we cite opioid analgesics that are µ-opioid agonists. Indeed, data on their efficacy in migraine are contradictory and they are associated with an increased risk of tolerance, physical dependence, and addiction although they continue to be prescribed extensively for acute migraine treatment in some parts of the world, particularly in the United State [124,125].

### 7.1. δ-Opioid Receptors

The δ-opioid receptors are widely expressed in brain regions involved in pain processing, such as the dorsal root and trigeminal ganglia, as well as the spinal cord [126]. However, unlike µ-receptors, their density is relatively low in nociceptive circuits of the midbrain and brainstem. In contrast, δ-opioid receptors are highly expressed in brain regions involved in processing the emotional contents of pain experience and mood regulation, including limbic forebrain structures, striatum, hippocampus, hypothalamus, and neuroendocrine system [127,128].

Preclinical studies in mice have shown that δ agonists inhibit mechanical and thermal allodynia induced by nitroglycerin (NTG) [129,130]. Indeed, mice lacking δ-opioid receptors show minimal modifications in acute pain threshold but exhibit significantly increased neuropathic and inflammatory pain [131,132]. Similarly, δ agonists have been found to effectively reduce behaviors that occur in response to persistent evoked pain [133]. Altogether, these findings suggest that, unlike m receptors, δ-opioid receptors have limited involvement in acute pain. 

There is converging evidence that δ-opioid receptors are implicated in anxiety, mood, and overall well-being as demonstrated by the fact that genetic deletion of the δ-opioid receptors or preproenkephalins results in increased levels of anxiety and depressive-like behaviors in animal models [134,135,136]. Similarly, pharmacological inhibition of δ-opioid receptors increases anxiety behaviors, which can be reversed by the δ-receptor agonist SNC80 [137].

Although, as abovementioned, δ-opioid receptors have only a partial role in acute pain, δ agonists have demonstrated efficacy in both acute and chronic models of migraine-associated allodynia [138]. Indeed, δ-agonists (e.g., SNC80, ARM390, and JNJ20788560) inhibit NTG-evoked mechanical hyperalgesia and SNC80 abolishes conditioned place aversion in mice and cortical spreading depression in response to continuous application of KCl in rodents [131]. Additionally, δ-opioid receptor agonists have been reported to inhibit the release of CGRP from activated trigeminal neurons [139]. Importantly, repeated treatment with SNC80 does not induce medication-overuse headache or opioid-induced hyperalgesia, in contrast to what happens administering morphine or sumatriptan, suggesting a lower potential for abuse using δ-agonists compared to µ-receptor agonists [140].

Since δ-opioid receptors are highly expressed in both the peripheral and CNS, it is unclear which sites regulate therapeutic effects on migraine. However, a recent study using a δ-opioid receptor knockout mice (e.g., for Nav1.8), which express about 50–70% less δ-opioid receptor in neurons of the dorsal root ganglia (considering that Nav1.8 is expressed in over 90% of peripheral nociceptors and mechanosensors), revealed that the anti-allodynic effect of SNC80 in the NTG model of migraine was only slightly reduced [141]. Nevertheless, the cephalic effects of SNC80 remained intact, as it continued to inhibit conditioned place aversion associated with NTG and decrease cortical spreading depression. These results suggest that the anti-migraine effects of δ agonists mainly occur in the CNS, indicating that brain-penetrant δ agonists would be more effective in treating migraine, although the activation and “functionalization” of the receptors are necessarily required.

In conclusion, based on their distribution in migraine-related structures in the nervous system and preclinical evidence, the involvement of δ-opioid receptors in migraine is undeniable and, considering the low abuse potential, they can certainly represent a target for migraine therapies [142]. In this regard, a δ agonist designed for migraine treatment (i.e., TRV250) has completed a Phase I clinical trial and has shown high safety and tolerability [143,144].

### 7.2. κ-Opioid Receptors

The κ-opioid receptors are widely expressed in structures involved in the modulation of reward, mood states, and cognitive function, such as the ventral tegmental area, nucleus accumbens, prefrontal cortex, hippocampus, striatum, amygdala, locus coeruleus, substantia nigra, dorsal raphe nucleus, and hypothalamus [145,146]. Therefore, κ-opioid receptors have recently been identified as a novel putative therapeutic target for the treatment of stress-related mood disorders, including anxiety, depression, and drug seeking [147]. Based on these observations, it has been hypothesized that κ-opioid receptors antagonists may serve as effective preventive therapies for stress-related migraine, as well as in patients with medication overuse headache.

Interestingly, a very recent study has demonstrated that systemic administration of a κ-opioid receptors agonist in rodents provoked behaviors, such as increased thirst and urination, which are consistent with clinically observed premonitory symptoms of migraine [148]. This finding suggests that targeting the hypothalamic κ-opioid receptors could enable early intervention for preventive treatment, even before the headache phase. In line with this hypothesis, it has been found that κ-opioid receptors blockade prevented both from stress-induced allodynia and increased plasma CGRP in rats chronically treated with sumatriptan as a model of medication overuse headache (making them hypersensitive to bright light stress and producing an increase in plasma CGRP leading to cephalic and peripheral allodynia) [149]. 

Moreover, a novel κ-opioid receptors antagonist, CERC-501 (formerly known as LY2456302) is currently in clinical trials for mood and anxiety disorders and has been shown to be safe and well-tolerated and selective orally available, reversible κ-opioid receptors antagonists are currently in development. Based on these early efficacy, safety and tolerability results, κ receptor antagonists may work as a promising therapy for migraine [150].

## 8. Transient Receptor Potential (TRP) Channel Family

The TRP channel superfamily comprises over 50 heterogeneous members, all of which serve as sensory transducers and contribute to various functions, such as thermo- and osmo-sensation, vision, touch, and pain [151,152]. Within mammals, this superfamily is composed of six subfamilies and 28 members, which act as nonselective cation-permeable channels. TRPs share a similar structural framework, consisting of six transmembrane domains that assemble as homo- or hetero-tetramers. TRP channels exhibit non-selective permeability to Ca^2+^, with different Ca^2+^/Na^+^ permeability ratios observed among the various members of the TRP superfamily. The six TRP subfamilies are classified as TRP canonical (TRPC), TRP melastatin (TRPM), TRP polycystin (TRPP), TRP mucolipin (TRPML), TRP vanilloid (TRPV), and TRP ankyrin (TRPA) channels [153]. Certain subfamilies encompass multiple members (e.g., the eight TRPM [TRPM1–8] receptors) or just one single member (i.e., the TRPA1 channel) and are influenced by an extensive array of endogenous and exogenous psychochemical stimuli, including intracellular mediators [152]. Several members of the TRP family are prominently expressed in distinct subsets of primary sensory neurons, where they play a role in detecting noxious physical and chemical stimuli. These channels, including TRPV1–4, TRPA1, and TRPM8, have collectively been referred to as thermo-TRP channels due to their ability to be activated by a broad range of temperatures, including noxious cold or heat [154]. Thermo-TRPs have been postulated to contribute to the transmission and modulation of nociceptive signals through multiple pathways, constituting the largest group of nociceptive ion channels involved in pain sensation [155]. Genetic and pharmacological studies have indicated that the TRPV1, TRPV4, and TRPA1 channels are major contributors in models of inflammatory and neuropathic pain. These receptors co-localize and are expressed in a subset of primary sensory neurons in the dorsal root ganglia, trigeminal ganglia, and vagal ganglia, but also in the hypothalamus (i.e., TRPV1 and TRPA1), as well as the cortex, caudate nucleus, and amygdala (i.e., TRPA1). Moreover, TRPA1 has been identified also in cells of the Schwann cell/oligodendrocyte lineage, keratinocytes, vascular endothelial cells, and skin fibroblasts [156]. 

Several TRPs have been implicated in different pathways involved in migraine [157]. Specifically, TRPA1-positive (TRPA1+) terminals have been observed in both C-fibers and Aδ-fibers of the trigeminal nucleus caudalis, frequently co-localized with CGRP, underpinning the release of neuropeptides from central and peripheral terminals of trigeminal nociceptors due to neuronal activation [158]. Meningeal vasodilation has been observed following the intranasal administration of TRPA1 agonists, while a selective TRPA1 receptor antagonist and a topical CGRP antagonist (CGRP8–37) have been shown to prevent the increase in blood flow [159]. In rats, a TRPA1 antagonist reduced mechanical allodynia caused by constriction of the infraorbital nerve and pain biomarkers triggered by NTG, likely inhibiting nitric oxide-mediated stimulation of TRPA1 in the soma of trigeminal nociceptors, with a release of reactive oxygen species (ROS) that, in turn, promotes a feed-forward ROS/TRPA1-dependent pathway that sustains allodynia [160,161,162]. Umbellulone, the primary component of Umbellularia californica (California bay laurel) and a TRPA1 agonist (able to induce nociceptive behavior, nasal mucosa vasodilation, and the release of CGRP from meningeal tissue in rats), has been found to trigger migraine-like and cluster-like headaches in susceptible individuals [163]. Interestingly, in mice knock-out for TRPA1 all the observed responses to umbellulone were eliminated. These findings are in line with hypothesis that migraine-inducing substances like chlorine, cigarette smoke, formaldehyde, and others may exert their effects in humans by stimulating TRPA1, where act also certain analgesics commonly used for the treatment of migraine attacks. Similarly, pyrazolone derivatives (e.g., dipyrone, antipyrine, aminopyrine, and propyphenazone), used to provide acute relief in migraine attacks with an unclear mechanism of action, have been found to selectively antagonize TRPA1 both in vitro and in vivo, attenuating nociception and allodynia in animal models of neuropathic and inflammatory pain through a TRPA1-mediated mechanism (independently from prostaglandin production) [164,165].

During the past decade, significant progress has been made in the development of selective and potent TRPA1 antagonists and five of them have been evaluated in clinical trials for their potential use in the treatment of pain or asthma with different results. However, TRPA1 antagonists have not been specifically tested for migraine so far although a series of high affinity and selective TRPA1 antagonists are currently undergoing phase I and phase II clinical trials for various diseases characterized by pain component [166].

## 9. Glutamate

Glutamate is a neurotransmitter that exerts a wide range of complex effects within the CNS through its binding to several different receptors, each possessing distinct structural and functional properties [167]. These receptors can be broadly classified into two groups: ionotropic glutamate receptors, which act as channels, and metabotropic glutamate receptors, which are coupled to G proteins [168]. The ionotropic receptors can be further categorized into N-methyl-D-aspartate (NMDA), α-amino-3-hydroxy-5-methylisoxazole-4-propionate (AMPA), and 2-carboxy-3-carboxymethyl-4-isopropenylpyrrolidine (kainate) receptors. On the other hand, the metabotropic receptors are divided into three groups (groups I–III), situated mainly post-synaptically and activate phospholipase C (I) or pre-synaptically and activate adenylyl cyclase (II–III) [169]. Distinct locations are also related to their differential functions (release or inhibition of glutamate) and thus their effects on neuronal activity. Group I receptors predominantly exert pronociceptive effects, whereas those in groups II and III exhibit predominantly antinociceptive properties. Various preclinical and clinical evidence have reported the role of glutamate in migraine pathophysiology, and drugs acting on this pathway have also been tested in these patients [170]. Indeed, kainate and glutamate receptor antagonists have demonstrated effectiveness in animal models of migraine, and the localization of glutamate receptors in regions associated with migraine pathophysiology, such as the trigeminal ganglion, trigeminal nucleus caudalis, and thalamus, further supports their potential involvement in migraine mechanisms [171].

Elevated levels of glutamate have been detected in patients with both episodic and chronic migraine, both during interictal and ictal period, in blood, saliva, as well as in cerebrospinal fluid [172,173,174,175,176,177]. Accordingly, migraine preventive treatments with distinct pharmacodynamics have been reported to reduce plasma glutamate levels, although these findings (especially blood measurements) have not been consistently replicated, and, to date, there is no conclusive pharmacological clinical evidence demonstrating the effectiveness of specifically targeting glutamate, or its receptors, as migraine treatment [178]. 

Several anticonvulsant medications used in migraine, with different recommendations, are known to interfere with the glutamate pathway [179], but the target-specific perampanel, an antagonist of AMPA receptors, failed to demonstrate efficacy in a phase 2 randomized controlled trial with a placebo conducted in 206 patients with migraine (NCT00154063). However, targeting glutamate or its receptors represents a therapeutic challenge due to their essential role in the normal functioning of the nervous system, which makes it difficult to act on without causing on-target adverse effects.

## 10. Evidence-Related Issues

Despite the large number of studies that have supported the identification of new potential targets for migraine prevention therapy over time, it should be emphasized that the related new drugs has also resulted in unexpected failures like in the latest striking case of the antibodies inhibiting PAC1 receptor of PACAP38 [46,180]. These results raise questions about the validity of migraine experimental models and study protocols from the preclinical models (in vitro and in vivo) through the to the provocation studies in humans. 

Among the several issues, the choice of the biological fluid to be examined (the role of plasma dosage of neuropeptides is highly debated and it is not clear whether the serum levels adequately reflect the activation of trigemino-vascular system), the body district where to carry it out, the phase of the migraine cycle to obtain the sample and, above all, the extreme methodological variability in the techniques used for the dosages [181,182,183,184]. 

Another methodological issue that worth it to be discussed regards animal models of migraine that have been developed and exploited efficiently in the last decades, ranging from the manipulation of the mouse genome to produce animals with human disease-like, through sensitive immunohistochemical methods to vascular, neurovascular and electrophysiological studies. Nevertheless, if, on one hand, they lead to better understanding potential migraine mechanisms, as well as the mechanism of action of migraine treatments, on the other hand, animal experimental models cannot explain all the features of migraine complexity as experienced by humans, surely remaining a major hurdle to overcome [185,186,187]. Indeed, it is noteworthy that migraine is a complex neurological disorder that primarily affects humans and, in animal models we can only focus on “proxy” of migraine attacks (such as allodynia, light aversion, pain behavior) that strongly limit the translatability of findings from preclinical models to human migraine. Moreover, leaving aside the obvious anatomical, physiological, and pharmacological differences between animals and humans, preclinical models typically tend to rely on inducing migraine-like episodes through experimental manipulations, by administering chemical agents or electrical stimulations with detrimental consequences in the understanding natural history of migraine, as well as the role of migraine triggers. The employment of animal models necessarily exclude specific pharmacological, cognitive and psychological elements (such as placebo or nocebo effects) which are known to play a critical role in migraine mechanisms, as well as in the treatment response in humans.

Finally, beyond the above mentioned limitation of animal models, caution is mandatory in the interpretation of data from human provocative studies, due to the fact that despite the similarities shared by provoked and spontaneous migraine attacks, substantial differences exist, mainly the source of migraine-inducing substances (exogenous vs. endogenous source) along with the absence of premonitory symptoms, underpinned by the different functional changes making the brain prone to an impending migraine attack [15,188]. In other terms, there is the high risk to focus, by inducing migraine-like attacks, on a snapshot of a very complex and multiphasic process.

## 11. Discussion

The increasing understanding of migraine pathophysiology strongly suggests that it is not only possible, but also advisable, to differentiate the pathophysiology of migraine as a condition of susceptibility of the CNS to certain trigger factors from the pathophysiology of migraine attacks as a result of episodic activation of the trigeminal vascular system [189]. 

Despite the highly variable nature of trigger factors, all potentially affecting the CNS, they converge to activate the trigemino-vascular system through mechanisms that remain unclear. Over the past few decades, therapeutic strategies have progressively shifted away from CNS mechanisms of migraine towards the identification of peripheral targets [190]. Among these, CGRP, linking central trigemino-cervical system activation with peripheral processes, like mast cell degranulation and dilation of meningeal arteries, has been identified as the major contributor to migraine pain [191]. However, there is still approximately 40% of patients with migraine that have demonstrated an inadequate response to monoclonal antibodies and the gepants-targeting CGRP pathway, raising the question of CGRP as the sole or the main contributor to the peripheral mechanisms underlying migraine attacks [7]. The response came from provocative studies that, since the first demonstration of the nitric oxide donor ability (i.e., glyceryl trinitrate) to induce more severe headaches in patients with migraine than in healthy volunteers, have highlighted and validated the role of numerous other molecules in migraine attacks ignition [15,192] (see Figure 1 and Table 1 for further information).

In line with this view, we cannot exclude future development of diagnostic criteria based on the response to drugs targeting specific molecules in order to classify patients with migraine according to the prevalent pathway involved, as a proxy of molecular-based diagnosis [193]. In other terms, whether the mechanisms underlying the pathophysiology of migraine in non-responsive patients are similar to those of responsive patients or whether different migraine phenotypes exist, each involving specific pathophysiological pathways (e.g., CGRP-related migraine vs. non-CGRP-related migraine), represent important avenues for ongoing and future research.

One could argue that targeting intracellular pathways shared by different neuropeptide signaling cascades might overcome the issue. Indeed, all the pathways activated by the different provocative molecules converge in a common intracellular downstream determinant where the second messengers cAMP or cGMP lead to the opening of potassium channels. The proof of the concept is in the ability of the KATP channel opener to achieve a migraine induction rate of 100% in patients with migraine patently higher than the administration of other provocative molecules primarily acting on specific extracellular upstream determinants (such as neuropeptides, channels, or receptors) [94].

Nevertheless, treatments targeting common intracellular downstream determinants, although promising as preventive antimigraine treatment, may raise safety concerns regarding the risks of off-target adverse effects associated with inhibiting shared and convergent sub-cellular pathways.

On the other hand, considering migraine as a ‘chronic brain disorder’ and migraine attacks as its ‘critical episodic manifestations’, it is worth it, considering whether drugs targeting extra-cerebral neuropeptides are treating the migraine disorder (in terms of brain predisposition to generate attacks) or merely inhibiting the peripheral compound of the trigemino-vascular system, whose ignition allows the clinical phenotype of migraine attacks, in a sort of peripheral “dam effect”. 

The latter concept may justify the recent observations of the frequency of migraine attacks worsening, after several months or years of treatment, when CGRP-mAbs are discontinued. In line with this interpretation, the findings from neurophysiological and neuroimaging studies, showing central effects of CGRP-mAbs treatment, may represent the epiphenomenon of the breakdown of the bombardment of supramedullary brain structures involved in sensory, affective, endocrine, and autonomic functions by intracranial pain signals originating in the meninges [194,195,196].

Furthermore, in a speculative way, the peripheral “dam effect” (with no modulation of brain dys-excitability) raises an important point in the interpretation of migraine throughout the lens of allostatic disease (characterized by structural, microstructural, and functional changes), in which the “sickness behaviour” during migraine attacks serves as a major alarm system, evolved over time, aimed at restoring the energy homeostasis in brains burdened by the maladaptive overload [197]. Therefore, future pharmacological research should focus on therapeutic strategies that not only target peripheral mechanisms, but also modulate central pathophysiological features of migraine brain. Probably, it is time to adopt a more holistic approach to the complexity of migraine, trying to address it as a comprehensive brain disorder with peripheral effectors in order to develop disease-modifying therapies with the primary future goal of treating underlying pathophysiological mechanisms, rather than merely managing symptoms.

## Figures and Tables

**Table 1:** Overview of neuropeptides, receptors, and ion channels described to be involved in migraine pathophysiology (other than CGRP).

Neuropeptide/ Receptor/Ion Channels	Chemical Structure	Receptors	Extra-CNS Action(s)	CNS Action(s)	Action(s) in Migraine	Provocative Studies in HC	Provocative Studies in Migraine
PACAP	Polypeptide with 2 isoforms: PACAP38 PACAP27	PAC1 VPAC1 VPAC2 MrgX2	Control of anterior pituitary hormone secretion Vasodilation Adrenaline and insulin secretion Immunosuppression	Regulation of neurotransmission Neuroprotective effect in response to: - Cerebral brain ischemia - Traumatic brain injury - Spinal cord injury	Dilation of dural and pial arteries Mast cells degranulation	IV 20 min administration induces migraine-like attack in 17% of HC	IV 20 min administration induces migraine-like attack in 65–75% of pts
VIP	Polypeptide of 28 amino acids	VPAC1 VPAC2 PAC1	Stimulation of gastrointestinal epithelial secretion and absorption Stimulation of heart contractility Vasodilation Glycogenolysis Anti-inflammatory and immune-modulatory	Neurotrophic effects Neurotransmission	Opening of potassium channels Dilation of dural and pial arteries	IV 25 min administration induces a marked vasodilation in the extracranial vessels and a very mild headache (42% of HC)	IV 2 h administration induces migraine-like attack in 71% of pts
Adrenomedullin	Polypeptide of 52/53 amino acids with 2 isoforms: AM1 AM2	AM1 receptor AM2 receptor	Vasodilation Bronchodilation Regulation of diuresis Regulation of food intake Gastrointestinal modulation Growth and hormone regulation Anti-inflammatory effects	Neuroprotective effect in response to: - Cerebral brain ischemia - Hemorrhagic stroke - Traumatic brain injury Pain processing modulation	Dilation of dural and pial arteries	NA	IV 20 min administration induces migraine-like attack in 55% of pts
Amylin	Polypeptide of 37 amino acids	AMY1 receptor AMY2 receptor AMY3 receptor	Delay of gastric emptying Reduction blood glucose by decreasing glucagon secretion	Inhibition of food intake	Pro-nociceptive action	NA	IV 20 min administration of amylin mimetic pramlintide induces migraine-like attack in 41% of pts
KATP channels	Octameric transmembrane protein complexes: 4 pore-forming Kir subunits and 4 SUR subunits	NA	Regulation of insulin secretion Control of vascular tone Protection against metabolic stress	Reduction in neurotransmitter release Neuronal protection from excitatory toxicity Analgesic and antinociceptive proprieties	Dilation of dural arteries	IV 20 min administration of 0.05 mg/min levcromakalim induces headache and MMA dilation reversed after sumatriptan 6 mg sc injection	IV 20 min administration of 0.05 mg/min levcromakalim induces migraine attack in 100% of pts with MwoA and 82% pts with MwA
BKCa channels	Octameric membrane protein complexes: 4 pore-forming subunits (α) and 4 accessory subunits (β)	NA	Control of vascular tone Regulation of cardiac rhythmicity Modulation erectile and urinary autonomic functions Regulation of gene expression and aging	Regulation of neurotransmitter release Modulation CBF Neuroprotection Modulate circadian rhythm	Control of vascular tone of cranial arteries Control of neuronal excitability	IV 20 min administration of 0.05 mg/min MaxiPost induces headache attacks (90% of HC)	IV 20 min administration of 0.05 mg/min MaxiPost induces migraine attacks in 100% patients with MwoA
Purinergic system	Adenosine ATP ADP	P1 (4) P2 - P2X (7) - P2Y (8)	Cell proliferation Cell death Inflammation Bone metabolism Bladder contraction Control of vascular tone Coagulation	Pain processing modulator Neurotransmission Neuromodulation	Vasodilation Neuronal sensitization	NA	IV 20 min administration of 120 µg/kg per minute adenosine induces headache attack in 78% of pts, no differences in migraine attack compared with placebo
δ-opioid receptors	GPCRs		NA	Regulation: Emotional behaviors Drug reinforcement Inhibitory controls Pain awareness and processing Learning memory	Regulation of acute pain threshold Inhibition of the release of CGRP from trigeminal neurons	NA	NA
κ-opioid receptors	GPCRs		NA	Modulation of: Reward Mood states Cognitive function	Implication in: Premonitory symptoms of a migraine Stress induced allodynia	NA	NA
TRP channel	6TM cation channel	6 subfamilies: - TRPC (1–7) - TRPM (1–8) - TRPP (1–3) - TRPML (1–3) - TRPV (1–6) - TRPA	Sensor of oxidative, nitrative and electrophilic stress Vasoconstriction Induction of vascular smooth muscle cell proliferation	Contribute to the transmission and modulation of nociceptive signals Detector of mechanical stimuli and noxious cold Mediation of vision, taste, olfaction, hearing and touch	Promotion of CGRP release Mediator in neurogenic inflammation Promotion of headache induced by toxic environmental irritants Modulation of vascular tone of meningeal artery	NA	NA
Glutamate	α-amino-acid anion	Ionotropic receptors: - NMDA - AMPA - Kainate Metabotropic receptors (groups I–III)	NA	Regulation of mood Contribute to neuroplasticity including: - LTP - Regulation of spine density - Synaptic reorganization	Primary role in CSD Facilitation of neuronal activity within the TNC Implication in peripheral and central neuronal sensitization	NA	NA

CNS: central nervous system; HC: healthy controls; PACAP: Pituitary Adenylate Cyclase-Activating Polypeptide; PAC1: Pituitary Adenylate Cyclase-Activating Polypeptide Type 1 Receptor; VPAC: Vasoactive Intestinal Polypeptide Receptor; IV: intravenous; min: minutes; pts: patients; VIP: Vasoactive Intestinal Polypeptide; h: hours; AM: Adrenomedullin; NA: not applicable; AMY: Amylin; KATP: Adenosine Triphosphate-Sensitive Potassium Channel; Kir: inward rectifier K+ channel; SUR: sulfonylurea receptor subunits; mg: milligrams; MMA: middle meningeal artery; sc: subcutaneous; MwoA: migraine without aura; MwA: migraine with aura; BKCa: Big Conductance Calcium-Activated Potassium Channel; CBF: cerebral blood flow; ATP: Adenosine Triphosphate; µg/kg: micrograms/kilos; GPCRs: G protein-coupled receptor; CGRP: Calcitonin Gene-Related Peptide; TRP: Transient Receptor Potential; TM: transmembrane; TRPC: Transient Receptor Potential Canonical; TRPM: Transient Receptor Potential Melastatin; TRPP: Transient Receptor Potential Polycystin; TRPML: Transient Receptor Potential Mucolipin: TRPV Transient Receptor Potential Vanilloid; TRPA: Transient Receptor Potential Ankyrin; NMDA: N-Methyl-D-aspartic acid; AMPA: α-amino-3-hydroxy-5-methyl-4-isoxazolepropionic acid; LTP: long term potentiation; CSD: cortical spreading depression; TNC: trigeminal nucleus caudalis.

## Data Availability

Not applicable.
